# Normative Hand Strength of Healthcare Industry Workers in Central Taiwan

**DOI:** 10.3390/ijerph18010187

**Published:** 2020-12-29

**Authors:** Victor Ei-Wen Lo, Shu-Min Chao, Hsin-Hung Tu

**Affiliations:** 1 Department of Occupational Safety and Health, China Medical University, Taichung City 40604, Taiwan; u104014409@cmu.edu.tw; 2 Department of Computer-Aided Industrial Design, Overseas Chinese University, Taichung City 40721, Taiwan; josephtu@ocu.edu.tw

**Keywords:** healthcare industry, hand strength, power grip, pinch, press, prediction

## Abstract

*Objectives*: The purpose of this study is to establish the norms of hand grip strength in the healthcare industry in Taiwan and propose models to predict the strength of hand movement by regression with demographic and anthropometric factors. *Methods*: This is a cross-sectional study with a stratified convenience sample of workers in healthcare service industries in central Taiwan. Three hundred twenty-nine healthy subjects were recruited. Strength of different hand movement were tested three times in both hands and rests were given between tests. *Results*: Female strength of these hand movement was 59.1% to 73.0% that in males (*p* < 0.001). In general, the hand strength of male workers in the healthcare industry was less than that of male workers in the manufacturing industry (*p* < 0.001). In the prediction model, sex and weight played important roles in predicting hand strength. *Conclusions*: The norms of different types of hand strength was investigated the first time in workers in the healthcare industry in Taiwan. The tasks performed by healthcare personnel vary widely, and this variable should be considered in a future prediction model.

## 1. Introduction

Hand strength is an important reference used in the medical diagnosis of hand disorders and injuries to evaluate and compare treatments or predict mortality [1,2,3,4,5,6,7,8,9,10,11,12,13]; it is also used in the assessment of the risk of musculoskeletal disorders (MSDs) for the improvement of task processes and design with regard to the manual handling of materials and other manual operations [14,15,16,17]. Therefore, the establishment of hand strength databases is an important issue in many countries.

Researchers have established hand strength databases for specific countries or ethnicities. Studies conducted in US measured grip strength [12,14,15,16] and pinch strength in adults [16]. In addition to measuring the grip strength in healthy adults [9,17], Harth et al. [18] measured grip, key, and palmar pinch strength in different jobs in Germany. Werle et al. [19] measured grip and pinch strength in a healthy adult Swiss population. Nilesn et al. [7] measured grip and pinch strength in an adult population in Norway. Harkonen et al. [20] measured grip strength in Finland. Adedoyin et al. [21] measured grip strength in healthy adults in Nigeria. In South Korean, Han et al. [22] measured grip and pinch strength, and Kim et al. [3] measured grip strength in adult populations. Yu et al. [13] measured grip strength in Chinese adults in Hong Kong. Su et al. [23] and Wu et al. [24] measured grip strength in the Chinese population in Taiwan. Most of the study participants in these previous studies were from the general population. There have been few studies focusing on individuals in specific industries. Schaub et al. [25] measured strength in the manufacturing industry employees in several EU countries. Lo et al. [26] measured grip and pinch strength among Taiwanese workers in the manufacturing industry.

In Taiwan, in view of the recent rapid emergence of the demand for healthcare services, MSDs problems have been an issue in the healthcare industry [27,28,29]. For examples, a radiologist or a radiologic technician needs to hold the probe when operated the ultrasound. A radiologist needs to press the syringe when giving the contrast media before the patients taking the resonance imaging, or computerised tomography scan. Dentists use root canal probe, spreader, condenser, endo activators to perform their jobs [30,31]. All of these tasks require different types of hand motions. Therefore, there is an urgent need for hand strength databases to be established for workers in the healthcare service industry that can be used for the evaluation of the risks of MSDs to inform the improvement of tasks and designs. However, it is unclear whether databases developed in previous studies can be directly adapted. Therefore, the study purposes were to (1) establish the norms of hand strength in the healthcare industry workers in Central Taiwan; and (2) propose models to predict the strength of hand movement by regression with demographic and anthropometric factors.

## 2. Materials and Methods

### 2.1. Study Subjects

This was a cross-sectional study with a convenience sample in the healthcare industry in central Taiwan. There were approximately four hundred forty-four thousand employees aged 15 to 64 years in the healthcare industry in Taiwan in 2017. We systematically sampled the 5 age strata with a 1 to 10,000 sampling rate due to the limited resources. Next, we calculated the sample size with the statistical power = 0.8, α = 0.05 with k-groups (9 age strata, and standard deviation = 9.7 kgw) using JMP 8.0 DOE function [32]. The results revealed that a total of 141 subjects were required and we should recruited at least 16 subjects for each age stratum. Therefore, we recruited more than 20 subjects of both sexes in each age stratum to increase the power. In total, 329 subjects participated in this study. All subjects were required to have worked in the healthcare industry for at least 6 months. The exclusion criteria included (1) any diagnosed MSDs in the past, including CTS, trigger finger, etc. in their upper extremities; and (2) any pain/discomfort in their upper extremities within 6 months; and (3) diseases affecting hand strength, e.g., rheumatoid arthritis or heart disease.

### 2.2. Instrumentation and Questionnaire

**Figure 1:**
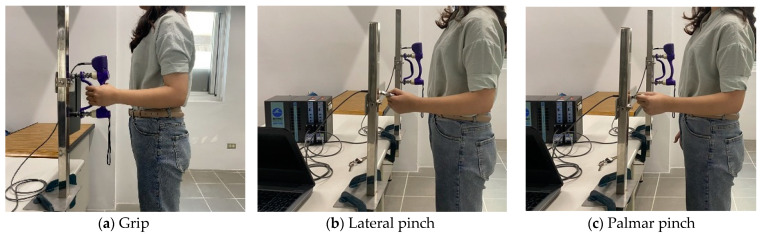

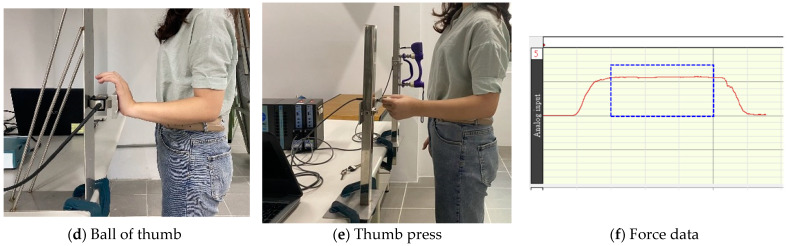
Illustrations of five types of hand movement and 5-s force data. (**a**) Grip; (**b**) Lateral pinch; (**c**) Palmar pinch; (**d**) Ball of thumb; (**e**) Thumb press; (**f**) Force data.

The detailed information on the instruments and experiment procedures was published in a previous study [29]. Grip strength (Figure 1a) was measured using a hand grip dynamometer (G200, Biometrics Ltd., Newport, UK). Pinch strength (Figure 1b,c) was measured using a pinchmeter (P200, Biometrics Ltd.) was used. The two devices were connected to a transducer amplifier (DA100C, BIOPAC System Inc., Goleta, CA USA). Finally, data with a sampling rate of 1000 Hz was collected and analyzed by an acquisition system and AcqKnowledge software (MP150, BIOPAC System, Inc.).

A customized system was designed to measure the pressing strength. An alumni cubic was attached to a load cell (LTZ-50KA, Kyowa, Japan), which was screwed to a height-adjustable L-shaped stainless steel stand (Figure 1d,e). The strength data was captured via a multifunction data acquisition device (USB-6002, National Instruments Co., Austin, TX, USA) with a sampling rate of 100 Hz and sent to a laptop.

Hand span was defined as the maximum distance between the tips of the thumb and little finger and measured using a Martin-type anthropometer. Then, the experimenter determine the optimal handle setting based on the Ruiz’s recommendations [31].

Demographic (age and sex), anthropometric (height, weight, hand width, hand span), and occupational information were collected with a 2-page questionnaire. To determine the dominant hand, the experimenter asked subject a question, “Which hand do you use for writing and eating?” Job information included the company’s, department information, title, and seniority in their current position.

### 2.3. Experiment Procedures

After instructions were explained to the participant, the experimenter started the test. During testing, the body postures of the subjects were aligned to the guideline by the American Society of Hand Therapists (ASHT). The subjects adducted their shoulders in a relaxed position, with their elbows at 90° flexion, and their wrists in neutral. The forearm was set to neutral for grip and pinch movements and pronated 90° for the pressing movements (Figure 1).

For each trial, the subjects were required to maintain the same movement for 5 s and the mean of 3 s represented the maximum strength for that trial (Figure 1e). The strength exerted during each type of hand movement in the right and left hand was measured 3 times. The mean value of three tests represented the maximum strength for a specific hand movement, which is the best approach with highest reliability from previous study [20,33,34,35]. The quality of the experiment data was determined by calculated the coefficient of variation (CV; defined as the mean value divided by the standard deviation multiplied 100). The experimenter asked a subject to perform the same movement again when the CV was greater than 10%. Then, the experimenter recalculated the coefficient of variation. Each subject performed the same type of hand movement at most five times to avoid muscle fatigue. Three-minute rest periods were taken between the two trials to reduce muscle fatigue [33]. All test trials were randomized and counterbalanced and took 60 min to complete the study.

### 2.4. Statistical Analysis

The means, standard deviations, and percentages represent the demographic and strength data. The Kolmogorov-Smirnov test for data distribution of normality was performed stratified by sex. The original data for grip strength and two types of pinch strength were normally distributed, but the data for the other two types of pressing motions were non-normally distributed. After data transformation using base-10 logarithms, the two types of press strength data had normal distributions. Statistical analyses of the ball of thumb and thumb press strength were performed with the transformed data, while the analysis of the other strength measurements were performed with the original data. To compare sex effect on the hand strength, *t*-tests were performed. A paired t test was performed to compare the effect of strength on handedness. To investigate the effects of age and movement types and their interactions on the strength, a repeated measures ANOVA was performed. The bonfferroni correction was used as a post hoc test. To investigate the relationship between the independent variables, Pearson’s correlation analysis was performed.

Stepwise multiple linear regression was used to explore the relationship of force pattern, sex, height, weight and age to establish a predictive model for muscle strength. The entry and removal probabilities were set at 0.05 and 0.10, respectively. Statistical significance was set as *p*-value < 0.05. SPSS Chinese version 22.0 (IBM Corporation, Armonk, NY, USA) was used for statistical analyses.

## 3. Results

### 3.1. Demographic Information of Study Subjects

In total, 329 healthy workers (162 males and 167 females) were recruited in the healthcare industry. Table 1 depicts the demographic and anthropometric results. There was a significant difference of body mass index between the sexes (*p* < 0.001). The mean BMI for males (24.8 ± 4.1 kg/m^2^) was in the “overweight” category as defined by the Ministry of Health and Welfare, Taiwan. With regard to hand width and hand span, males had significantly wider hands than females (*p* < 0.001). There were only 8 females (2.4%) who selected the second handle as their optimum grip span. The third handle as the optimum grip span was selected by 97.4% of the females and all males. Ninety-five percent of the participants were right-handed. Therefore, we reported the results based on the analysis of laterality instead of handedness. Regarding education levels, more than 66% of the participants had received undergraduate and graduate training, which was not surprising due to the unique characteristics of the healthcare industry.

### 3.2. Effect of Sex

Table 2 shows that there were significant differences between the sexes in all five types of hand movements (*p* < 0.001), and the following statistical analyses of the strength data were stratified by sex. The five strength measurements in females were 59.1–73.0% of the values in males.

For both sexes, the grip strength, lateral and palmar pinch strength for the right hand were significantly stronger than the values for the left hand. No significant strength difference in the ball of thumb was observed for both sexed.

### 3.3. Hand Movements and Age Effects on Hand Strength

To investigate the effects of age and types of hand movements on strength, we performed repeated-measures ANOVA. Since the results of Mauchly’s test was significant (Table S1), the Huynh-Feldt method was used to determine the significance. Among the males, the types of hand movements mainly affected strength for both right hand (*p* < 0.001, η^2^ = 0.938) and the left hand (*p* < 0.001, η^2^ = 0.98) (Table S1). Post hoc tests showed that grip strength was significantly greater than the two types of pinch strength, while the lateral pinch strength was 1.1 kgw and 0.86 kgw greater than the palmar pinch strength for the right hand and left hand (*p* < 0.001), respectively. There was a main effect of age on the strength for both right hand (*p* < 0.001, η^2^ = 0.123) and left hands (*p* < 0.001, η^2^ = 0.131). The strength in participants aged 25–54 years was greater than that in participants aged 20–24 years and 55–64 years for both hands. Furthermore, there were interactions between the types of hand movements and age for the right hand (*p* = 0.002, η^2^ = 0.101) and the left hand (*p* = 0.003, η^2^ = 0.094).

Among the females, the types of hand movements mainly affected the hand strength for the right (*p* < 0.001, η^2^ = 0.939) and the left hand (*p* < 0.001, η^2^ = 0.946). Similar to the results of the males, the grip strength for females was greatest and greater than the pinch strength, while the lateral pinch was 0.42 kgw and 0.27 kgw significantly greater than the palmar pinch for the right and left hand, respectively (all *p* < 0.001). There was an age effect on the strength for the left hand (*p* = 0.034, η^2^ = 0.062). Post hoc tests revealed that the strength of participants aged 35–54 years was greater than that of participants aged 20–24 years.

Regarding the ball of thumb and thumb press, the types of hand movement affected the strength on both hands for both sexes (all *p* < 0.001, η^2^: 0.599~0.673) after the data was log-transformed (Table S2). The ball of the thumb strength was 1.3~1.4 kgw greater than that of the thumb press regardless of sex or hand laterality. However, there was no main effect of age nor was there an effect of the interaction of types of movements and age.

The grip strength of the male participants reached its maximum (41.1 ± 8.1 kgw) in the group aged 35–44 years and decreased in the right hand as age increased (Table 3). For the right hand, the grip strength in the participants aged 20–24 years was significantly lower than that in those aged 25–34 and 35–44 years (*p* = 0.036 and *p* = 0.001, respectively). For the left hand, the grip strength in the participants aged 20–24 years was significantly lower than that in those aged 35–44 and 45–54 years (*p* = 0.003 and *p* = 0.048, respectively), and the grip strength in the participants aged 35–44 years was significantly higher than that in those aged 55–64 years (*p* = 0.029). In the female participants, the grip strength also reached its maximum in the group aged 35–44 years (23.0 ± 5.2 kgw) and decreased in the right hand as age increased (Table 4). There was no significant difference in either hand among the five age groups.

The strength in the ball of the thumb on the right hand in male participants reached its maximum on the right hand (7.3 ± 1.3 kgw) and left hand (7.5 ± 1.3 kgw) in the group aged 45–54 years, but there were no significant differences among the age groups (*p* = 0.953 for and *p* = 0.809 for left hand, respectively). Among female participants, the ball of the thumb strength reached its maximum on the right hand (5.0 ± 1.4 kgw) and on the left hand (5.2 ± 1.3 kgw) in the group aged 55–64 years. For the left hand, the maximum strength was 5.2 ± 1.3 kgw in the group aged 55–64 years. Again, there were no significant differences among the age groups for right and left hands (*p* = 0.815 and *p* = 0.595, respectively).

There were no effects of age on the strength of the thumb press with regard to sex or laterality. The thumb press strength for the right hand for males reached its maximum in the group aged 45–54 years (6.0 ± 1.3 kgw). The maximum strength was 6.0 ± 1.4 kgw in the group aged 45–54 years for the left hand. For females, the thumb press strength reached its maximum in the group aged 55–64 years for the right hand (3.9 ± 1.4 kgw) and the left hand (3.9 ±1.4 kgw).

The lateral pinch strength for the right hand of the male participants reached its maximum (8.4 ± 1.7 kgw) in the group aged 25–34 years, and there was a significant difference among all age groups (*p* < 0.001). The lateral pinch strength in the group aged 20–24 years was significantly lower than that in those aged 25–34, 35–44 and 45–54 years (*p* < 0.001; *p* = 0.016; *p* = 0.002). For the left hand, the lateral pinch strength in the group aged 20–24 years was significantly lower than that in those aged 25–34, 35–44 and 45–54 years (*p* < 0.001; *p* = 0.019; *p* = 0.001). Among female participants, the lateral pinch strength for the right hand reached its maximum (5.6 ± 1.0 kgw) in the group aged 45–54 years. For the left hand, the lateral pinch strength in the group aged 20–24 years was significantly lower than that in those aged 35–44 and 45–54 years (*p* = 0.031; *p* = 0.040).

The palmar pinch strength in the male participants reached its maximum (7.1 ± 1.4 kgw) in the group aged 45–54 years for the right hand, and there was a significant difference among all age groups (*p* = 0.011). The palmar pinch strength in the group aged 20–24 years was significantly lower than that in those aged 25–34 and 45–54 years (*p* = 0.028; *p* = 0.016). For the left hand, the palmar pinch strength in the group aged 20–24 years was significantly lower than that in those aged 25–34 and 45–54 years (*p* = 0.029; *p* = 0.002). The palmar pinch strength in the female participants for the right hand peaked in the group aged 25–34 and 45–54 years, but there were no significant differences among the age groups (*p* = 0.666). For the left hand, the maximum palmar strength was in the group aged 45–54 years (5.0 ± 0.8 kgw). There was no effect of age on the palmar strength of the right and left hand (*p* = 0.666 and *p* = 0.216, respectively).

### 3.4. Correlation Coefficients

The strength measurement in both hands were strongly correlated, with coefficients ranging from 0.876 to 0.947 (Table S3). Hand strengths was positively correlated with height, weight, BMI, hand width and hand span (*p* < 0.001). Sex was highly associated with grip strength for both hands and lateral pinch strength for the right hand; sex was moderately correlated with palmar strength, ball of the thumb strength and thumb press strength for both hands and lateral pinch strength for the left hand (*p* < 0.001). Height and weight were moderately correlated with all types of hand strengths (r = 0.525~0.669, *p* < 0.001; r = 0.481~0.625, *p* < 0.001). However, all types of hand strengths were also moderately correlated with hand width and hand span (r = 0.438~0.631, *p* < 0.001; r = 0.454~0.575, *p* < 0.001).

### 3.5. Occupation Effects on the Hand Strength

We compared the hand strength of workers in the healthcare and manufacturing industries in Taiwan [26]. There were significant differences in grip strength, ball of thumb strength, thumb press strength and palmar pinch strength in both hands between the different occupations among males (Table 5). For the females, there were significant differences in ball of thumb, thumb press and palmar pinch strengths in both hands between the different occupations. The hand strength of manual workers was significantly greater than that of healthcare personnel. For males, the effect of occupation was significant for all five different types of strength, but for females, only three types of strength were significantly different.

### 3.6. Prediction Models

Prediction models based on demographic information (e.g., age, sex) and anthropometric data (e.g., weight, height) for different types of hand strength in the right and left hands are shown in Table 6. The grip strength prediction model for the right hand that included sex, height and weight had the best explanatory power (adjusted R^2^ = 0.598), and the prediction model for the left hand that included sex, weight, BMI and age had the best explanatory power (adjusted R^2^ = 0.586). The lateral pinch strength of the right hand was best predicted by the model including sex, age, height and weight (adjusted-R^2^ = 0.567); for the left hand, the prediction model with sex, weight and age had the best explanatory power (adjusted-R^2^ = 0.551). Sex and weight were the only two variables that were predictive of the maximum palmar pinch strength, and the predictive power was the worst for this type of hand strength (adjusted-R^2^ = 0.399–0.434). After base-10 log-transformation, the ball of thumb and thumb press strength data had a normal distribution. Sex and weight were predictive of the ball of thumb and thumb press strength for both hands, and the adjusted-R^2^ ranged from 0.407 to 0.486. Sex and weight played important roles in predicting hand strength.

The models including demographic and anthropometric variables explain less than 60% of the variance and are therefore inadequate. Previous studies showed that there were good correlations of between the right and left hand strength for the same type of hand movement. Therefore, we proposed models to predict the strength of one hand that incorporated the strength of the opposite hand. Table 7 shows that using the strength of one hand to predict the strength of the other improved the explanatory power (adjusted-R^2^ = 0.773–0.951). However, the only important variable was sex, which was included in only 6 of 10 equations.

Repetitions of the tests of hand strength using similar muscle groups may result in muscle fatigue. The best way to minimize muscle fatigue is to reduce the number of hand movements. In addition, grip strength is widely used in many fields, e.g., ergonomics, physical therapy, rehabilitation, occupational epidemiology, and medicine. Therefore, we include grip strength as a predicting variable in the model and the results are shown in Table 8. The motions of gripping, lateral pinching and palmar pinching use similar muscle groups. The prediction model for lateral pinch strength in the right hand that included grip strength had the best explanatory power (adjusted-R^2^ = 0.679), and the palmar pinch prediction model that included grip strength had a lower explanatory power (adjusted-R^2^ = 0.559) than the model that included lateral pinch strength in both hands. However, the predictive models for the ball of thumb and thumb press strength had weaker explanatory power (adjusted-R^2^ =0.497 –0.436). The tests for the strength of the thumb press and ball of thumb use similar muscle groups. The adjusted R^2^ values for the models using the ball of thumb strength to predict the thumb press strength were 0.741 and 0.698, which were larger than those for the models using grip strength.

## 4. Discussion

Among the strength of five hand movements measured in employees in the healthcare industry, the order ranked from strongest to weakest for the right hand was as follows: grip, lateral pinch, ball of thumb, palmar pinch, and thumb press. As mentioned in previous studies, grip strength uses the muscles of the forearm, so it exerts the strongest force of the five different types, and the lateral pinch force is greater than the palmar pinch force [5,6,26]. Fewer muscle groups are used to perform the thumb press than the ball of thumb press, and the force exerted is consequently smaller.

Consistent with the findings of several previous studies, the results of this study showed that hand strength in both hands were significantly greater in males than in females [2,5,7,8,9,10,11,12,13,19,24,26,36,37,38]. Female hand strength was between 59.1% and 73% of male hand strength for both hands. Mahammadian studied Iranian adults and found that the maximum strength in females was approximately 60.1% (26.5/44.1 kgw) that of males [6]. Steiber found the grip strength of females to be approximately 64% (33.3/52 kgw) that of males in Germany [9]. With regard to lateral pinch strength, Werle et al. studied Swiss adults and found that the pinch strength of females was approximately 69.2% (7.2/10.4 kgw) that of males [19]. Han et al. found that the palmar pinch strength of females was approximately 67.7% (6.7/9.9 kgw) that of males [22]. There are some reasons to explain the strength difference between males and females, including muscle fibre specificity; fat, bone and muscle composition; and exercises [39,40,41,42]. As mentioned above, we also found that most of the measurements of hand strength in Taiwanese healthcare practitioners were lower than those in other countries.

Age is one of the factors affecting hand strength [9,10,19,43,44]. Among the five age groups, the grip strength and pinch strength of males differed significantly, and the pinch strength of females differed significantly. The grip strength reached its maximum in the group aged 35–44 years (41.1 ± 8.1 kgw) for males and declined after 54 years of age. For females, the grip strength reached its peak in the group aged 35–44 years (23.0 ± 5.2 kgw). The results were the same as those of a previous study in Taiwan, and the maximum grip strength of males was in the group aged 30–39 years old and that of females was in the group aged 40–49 years old [23]. The study by Lim et al. showed that the maximum grip strength in males was in the group aged 30–39 years, while that in females was in the group aged 40–49 years [4]. Han et al. investigated the lateral pinch, palmar pinch, and grip strength and found that the hand strength of Korean men and women peaked in the group aged 30–39 years [22]. The peak lateral pinch strengths in this study were in the group aged 25–34 years for males and the group aged 35–44 years for females (8.4 ± 1.7 and 5.1 ± 1.1 kgw, respectively). For press strength, there were no significant differences for either sex.

Comparisons were made between the healthcare workers and manufacturing workers. Ball of thumb strength, thumb press strength and palmar pinch strength for healthcare workers were significantly smaller in both hands than that for manufacturing workers among males and females. However, there were significant difference in grip strength between healthcare workers and manufacturing workers among males, but not among females. The results in the present study agree with the results of the study by Eksioglu [2], which showed that there were no differences in grip strength among females when comparing manual workers to nonmanual workers. However, our study results contradicted the results of that study with regard to grip strength in males [2]. Liu and Chu demonstrated the training effect on the job demands and their study results revealed that retired workers who had previously worked as construction site workers had greater grip strength than those who had previously worked as office workers [45]. However, Hossain et al. showed that the grip strength of office workers (31.2 ± 8.1 kg) was significantly greater than that of manual workers (28.7 ± 8.0 kg) and unemployed people (26.3 ± 8.6 kg) among males, while there was no significant difference among females [37]. The possible reason to explain the difference in the effect of occupation is the difference in physical demands. Based on our experience when visiting the manufacturing facilities, tasks usually require higher physical demands and are assigned to males. Women are mainly responsible for the tasks with a lower physical demands, such as quality assurance or quality control. Furthermore, there are relatively fewer high job demanding tasks in the healthcare industry than in the manufacturing industry. Therefore, the grip strength among male manufacturing workers was greater than that among the male healthcare workers.

Sex and weight are the two important variables that can be used to predict hand strength. As in the previous study by Hanten et al. [15], there were good correlations between the measurements of strength in the right and left hands for the same type of hand movements (both hands; adjusted-R^2^ = 0.87). Including the grip strength of the right hand in the predictive models for other types of hand movements yielded higher correlation coefficients than using only demographic information and anthropometric data. With regard to grip strength, the prediction model developed here explains more variance (adjusted-R^2^ = 0.598–0.586) than the earlier models presented by Wang et al. [12]. The adjusted-R^2^ of the models in Wang’s study ranged from 0.32 to 0.44. The prediction models in other studies had greater explanatory power than those in the present study [4,6,8,11]. This was true not only for grip strength but also for pinch strength. The explanatory power of the pinch strength model was weaker in this study than in previous studies [6]. The tasks performed by medical service personnel varies widely, but this variation was not taken into account in the prediction model. Few studies have developed models including the grip strength of the right hand as an explanatory variable, relying on other demographic and anthropometric variables to predict the other types of hand strength. Using the grip strength of the right hand increased the explanatory power, especially for lateral pinch and palmar pinch strength. The models that included the ball of thumb strength of right hand in addition to demographic and anthropometric variables were better able to predict thumb press strength. Because the ball of thumb and thumb press strength tests use similar muscle groups, the explanatory power was stronger than that of the model including grip strength.

There are some strengths in this study. First, all study participants were recruited from the healthcare industry in central Taiwan. The results can be applied when making recommendations regarding healthcare jobs. Second, the prediction model made it possible to predict five different types of hand strength in healthy healthcare personnel in Taiwan. There are also some limitations. First, hand strength was measured in healthy healthcare industry workers, and the results cannot be generalized to those with MSDs affecting the upper limbs. The inability to generalize the developed grip strength equations to populations involving different ethnic groups, occupations, and types of tasks is another limitation of this study. Lo et al. already reported the same types of hand motions in the manufacturing industry [26]. Future studies are needed to include other industries. For example, constructions and retails which rank the top three industries. There are other anthropometric dimensions, such as hand circumference, arm circumference, thumb circumference, and shoulder to elbow length, as well as other supplementary variables (e.g., types of sport) that could influence grip strength that were not taken into account in this study. For example, Su et al. showed that thumb length positively correlated to the palmar and lateral pinch strength, and index finger length was negatively correlated to the palmar and lateral pinch strength [23]. Another study showed that hand length and forearm length were strongly correlated with pinch strength [6]. Finally, we did not report the strength stratified by job titles and age, which may confound the analysis stratified by sex. For example, more nurses and nurse assistants were females (76.7% were females), while members of the maintenance department and EMT were predominantly males (all males). For males, the mean age of male maintenance workers (43.0 ± 10.5 years) were significantly greater than the mean age of male nurses (31.3 ± 11.0 years) and other male volunteers (38.5 ± 13.0 years). The grip strength among male maintenance workers was 1.5 kgw greater than the strength of male nurses. The mean age of female nurses was significantly younger than the mean age of other female volunteers (35.8 ± 12.4 years vs. 42.2 ± 12.8 years). The strength of ball of thumb press in the right hand for female nurses was significantly smaller than the strength of other female volunteers. Future studies are needed to investigate the differences stratified by job title and age.

## 5. Conclusions

This study was the first to investigate the norms of strength among five different types of hand movements in the healthcare industry in Taiwan. The strength measured in females for these five types of hand movements was 59.1%–73.0% that in males. For both sexes, there was a main effect of the types of hand strength for the right hand and the left hand. In the prediction model, sex and weight were the two most important variables for predicting hand strength. The tasks performed by healthcare personnel vary widely, and this variable should be considered in future prediction models.

## Figures and Tables

**Table 1:** Demographic information of the subjects in the healthcare industry (*n* = 329).

Variables	Males (*n* = 162)	Females (*n* = 167)	*p*-Value ^1^	Total (*n* = 329)
	Mean ± Stdev *n*(%)	Mean ± Stdev *n*(%)		Mean ± Stdev *n*(%)
Age (years)	38.0 ± 12.7	38.7 ± 12.9	*p* = 0.617	38.3 ± 12.8
Height (cm)	171.7 ± 6.7	159.2 ± 5.0	*p* < 0.001 **	165.3 ± 8.6
Weight (kg)	73.3 ± 13.9	57.6 ± 9.0	*p* < 0.001 **	65.3 ± 14.0
BMI (kg/m^2^)	24.8 ± 4.1	22.8 ± 3.5	*p* < 0.001 **	23.8 ± 3.9
Hand width (cm)	8.8 ± 0.7	7.8 ± 0.5	*p* < 0.001 **	8.3 ± 0.8
Hand span (cm)	20.5 ± 1.3	18.3 ± 1.2	*p* < 0.001 **	19.4 ± 1.7
Handle setting(count)				
2	0(0%)	8(4.8%)		8(2.4%)
3	162(100%)	159(95.2%)		321(97.6%)
Dominant hand (count)				
R	152(93.8%)	161(96.4%)		313(95.1%)
L	10(6.2%)	6(3.6%)		16(4.9%)
Education				
Junior high school or less	5(3.1%)	7(4.2%)		12(3.6%)
Senior high school	15(9.3%)	21(12.6%)		36(10.9%)
College	22(13.6%)	41(24.6%)		63(19.1%)
University or graduate school	120(74.1%)	98(58.7%)		218(66.3%)
Exercise				
None	20(12.3%)	40(24.0%)		60(18.2%)
Sometimes	39(24.1%)	60(35.9%)		99(30.1%)
1–2 times per week	24(14.8%)	19(11.4%)		43(13.1%)
3–4 times per week	63(38.9%)	41(24.6%)		104(31.6%)
>5 times per week	16(9.9%)	7(4.2%)		23(7.0%)

Note: ^1^: students’ *t*-test. **: *p* < 0.001.

**Table 2:** Statistics summary of strength by type of hand movement, sex, and hand laterality (Unit: kgw).

Type of Hand Movement	Laterality	Males (*n* = 162)							Females (*n* = 167)							Female/MaleRatio ^2^
		Mean ± SD	Max.	90% Tile	50% Tile	10% Tile	Min.	*p*-Value ^1^	Mean ± SD	Max.	90% Tile	50% Tile	10% Tile	Min.	*p*-Value ^1^	
Grip	R	37.2 ± 8.5	64.1	48.2	36.3	26.8	17.9	*p* < 0.001 **	22.0 ± 4.8	33.3	28.0	22.4	15.5	10.0	*p* < 0.001 **	59.1%
	L	34.6 ± 8.0	64.3	43.9	33.5	24.6	17.0		21.2 ± 4.5	30.7	27.7	21.1	14.9	11.5		61.2%
Ball of Thumb	R	7.2 ± 1.3	17.0	9.9	7.1	5.3	3.6	*p* = 0.863	4.9 ± 1.3	10.0	7.0	5.0	3.1	2.2	*p* = 0.363	68.1%
	L	7.2 ± 1.3	17.0	9.9	7.2	5.0	3.8		4.9 ± 1.3	11.0	7.0	5.0	3.5	2.6		68.1%
Thumb Press	R	5.7 ± 1.3	12.0	8.1	5.9	4.0	2.9	*p* = 0.236	3.7 ± 1.3	7.5	5.2	3.9	2.4	1.5	*p* = 0.050 *	64.9%
	L	5.6 ± 1.3	13.0	8.3	5.6	3.9	2.0		3.6 ± 1.4	7.8	5.1	3.8	2.2	1.5		64.2%
Lateral Pinch	R	7.9 ± 1.5	11.4	9.8	7.9	5.9	4.6	*p* < 0.001 **	5.3 ± 1.1	8.8	6.7	5.3	4.0	2.4	*p* < 0.001 **	67.1%
	L	7.2 ± 1.5	11.1	9.1	7.2	5.3	4.0		4.8 ± 1.0	7.7	6.1	4.7	3.7	2.4		66.7%
Palmar Pinch	R	6.8 ± 1.4	10.7	8.6	6.8	5.1	3.7	*p* < 0.001 **	4.9 ± 1.0	8.8	6.2	4.9	3.5	2.7	*p* < 0.001 **	72.1%
	L	6.3 ± 1.2	9.0	8.1	6.4	4.8	3.4		4.6 ± 0.9	7.2	5.7	4.5	3.5	2.3		73.0%

Note: ^1^: Paired *t*-test.; ^2^: Student’s *t*-test and all *p* < 0.001 *: *p* < 0.05; **: *p* < 0.001.

**Table 3:** Summary of Strength data by age, type of hand movements and hand laterality among males (*n* = 162, unit: kgw).

Type of Hand Movement	Age Group (n)	20~24 (36)		25~34 (38)		35~44 (33)		45~54 (35)		55~64 (20)		*p*-Value
	Hand Laterality	Mean(SD)	Min-Max	Mean(SD)	Min-Max	Mean(SD)	Min-Max	Mean(SD)	Min-Max	Mean(SD)	Min-Max	
Grip	Right	33.4 (7.7)	21.4–52.4	38.8 (9.6)	17.9–60.9	41.1 (8.1)	27.5–64.1	37.3 (7.2)	22.7–52.6	34.2 (7.2)	30.3–47.9	*p* = 0.001 **
	Left	31.0 (6.9)	17.0–49.7	35.9 (8.9)	19.1–64.3	37.7 (8.2)	24.9–61.2	36.0 (6.6)	24.3–53.4	31.3 (6.6)	20.8–42.8	*p* = 0.001 **
Ball of Thumb	Right	7.2 (1.3)	4.0–11.1	7.0 (1.4)	3.8–14.2	7.2 (1.2)	5.4–10.0	7.3 (1.3)	4.0–16.8	7.2 (1.3)	5.4–12.7	*p* = 0.953
	Left	7.2 (1.3)	4.1–11.4	7.0 (1.4)	3.6–14.6	7.1 (1.2)	5.0–11.1	7.5 (1.3)	4.4–17.3	7.0 (1.3)	4.4–10.8	*p* = 0.809
Thumb Press	Right	5.7 (1.3)	3.3–9.0	5.5 (1.4)	2.9–10.7	5.6 (1.3)	3.4–9.5	6.0 (1.3)	3.8–11.6	5.7 (1.3)	3.2–9.5	*p* = 0.737
	Left	5.6 (1.3)	2.8–9.9	5.5 (1.4)	2.5–10.9	5.4 (1.3)	2.9–9.1	6.0 (1.4)	3.5–12.7	5.8 (1.3)	3.1–9.0	*p* = 0.689
Lateral Pinch	Right	7.0 (1.4)	4.6–9.8	8.4 (1.7)	5.1–11.4	8.1 (1.5)	5.5–11.1	8.3 (1.5)	5.4–11.3	7.6 (0.7)	5.9–8.7	*p* < 0.001 **
	Left	6.3 (1.4)	4.0–9.3	7.8 (1.6)	4.0–11.1	7.4 (1.7)	5.0–11.0	7.6 (1.1)	5.0–9.4	6.7 (1.0)	5.1–9.0	*p* < 0.001 **
Palmar Pinch	Right	6.1 (1.6)	3.7–9.2	7.0 (1.3)	4.1–9.3	7.0 (1.4)	4.3–10.7	7.1 (1.4)	4.9–10.1	6.7 (0.9)	4.9–8.0	*p* = 0.011 *
	Left	5.7 (1.1)	3.4–8.1	6.5 (1.3)	3.4–9.0	6.5 (1.3)	4.1–8.9	5.9 (1.0)	4.7–8.7	5.9 (1.0)	3.4–8.1	*p* = 0.001 *

Note: * *p* < 0.05; ** *p* < 0.001.

**Table 4:** Summary of Strength data by age, type of hand movements and hand laterality among females (*n* = 167, unit: kgw).

Type of Hand Movement	Age Group (n)	20~24 (35)		25~34 (36)		35~44 (34)		45~54 (32)		55~64 (30)		*p*-Value
	Hand Laterality	Mean(SD)	Min-Max	Mean(SD)	Min-Max	Mean(SD)	Min-Max	Mean(SD)	Min-Max	Mean(SD)	Min-Max	
Grip	Right	21.1 (4.1)	13.1–28.8	21.8 (4.9)	11.9–33.3	23.0 (5.2)	13.1–32.5	22.3 (5.2)	11.8–32.1	21.7 (4.3)	10.0–33.1	*p* = 0.525
	Left	19.7 (4.2)	11.8–29.8	20.4 (4.3)	13.0–29.5	22.2 (4.5)	12.6–30.6	22.3 (4.9)	11.5–30.1	21.6 (4.3)	11.6–30.7	*p* = 0.065
Ball of Thumb	Right	4.7 (1.3)	2.5–8.3	5.0 (1.3)	2.7–7.7	4.8 (1.3)	3.2–7.2	4.8 (1.3)	2.9–8.6	5.0 (1.4)	2.2–9.6	*p* = 0.815
	Left	4.7 (1.3)	2.6–7.6	5.1 (1.3)	3.1–8.0	4.8 (1.2)	3.5–7.4	4.8 (1.3)	2.7–8.8	5.2 (1.3)	3.1–10.5	*p* = 0.595
Thumb Press	Right	3.6 (1.4)	1.7–7.5	3.8 (1.4)	1.8–5.7	3.6 (1.3)	2.0–6.1	3.5 (1.4)	1.5–5.4	3.9 (1.4)	1.6–6.3	*p* = 0.544
	Left	3.4 (1.4)	1.6–6.7	3.7 (1.3)	2.1–6.1	3.5 (1.3)	2.0–5.7	3.5 (1.4)	1.6–6.2	3.9 (1.4)	1.5–7.8	*p* = 0.276
Lateral Pinch	Right	4.9 (0.9)	2.4–7.1	5.1 (0.8)	3.5–6.6	5.5 (1.2)	3.3–8.0	5.6 (1.0)	2.7–7.9	5.4 (1.3)	3.3–8.8	*p* = 0.042 *
	Left	4.5 (0.8)	2.7–6.6	4.7 (0.9)	3.0–6.3	5.1 (1.1)	3.6–7.7	5.1 (1.0)	2.6–7.3	4.8 (1.1)	2.4–6.8	*p* = 0.014 *
Palmar Pinch	Right	4.7 (0.9)	2.7–6.5	5.0 (1.1)	2.8–8.8	4.9 (1.0)	2.8–6.7	5.0 (0.8)	3.0–6.5	4.9 (1.3)	2.8–7.6	*p* = 0.666
	Left	4.3 (0.8)	2.6–6.5	4.5 (0.9)	2.9–7.2	4.6 (0.7)	3.0–6.4	4.8 (0.8)	3.0–6.1	4.6 (1.1)	2.3–6.4	*p* = 0.216

Note: * *p* < 0.05.

**Table 5:** Effect of occupation on strength stratified by sex, types of hand movement, and laterality.

Sex	Type of Hand Exertion	Hand Laterality	Healthcare Service (*n* = 162)	Manufacturer (*n* = 99)	Ratio	*p*-Value ^1^
			Mean ± SD	Mean ± SD		
Male	Grip	R	37.2 ± 8.5	41.9 ± 9.4	88.8%	*p* < 0.001 *
		L	34.6 ± 8.0	39.7 ± 8.4	87.2%	*p* < 0.001 *
	Ball of Thumb	R	7.2 ± 1.3	10.4 ± 1.4	69.2%	*p* < 0.001 *
		L	7.2 ± 1.3	10.6 ± 1.4	67.9%	*p* < 0.001 *
	Thumb Press	R	5.7 ± 1.3	8.2 ± 1.4	69.5%	*p* < 0.001 *
		L	5.6 ± 1.3	8.0 ± 1.4	70.0%	*p* < 0.001 *
	Lateral Pinch	R	7.9 ± 1.5	8.7 ± 1.6	90.8%	*p* < 0.001 *
		L	7.2 ± 1.5	8.2 ± 1.7	87.8%	*p* < 0.001 *
	Palmar Pinch	R	6.8 ± 1.4	8.0 ± 1.7	85.0%	*p* < 0.001 *
		L	6.3 ± 1.2	7.4 ± 1.7	85.1%	*p* < 0.001 *
Female	Grip	R	22.0 ± 4.8	21.8 ± 5.1	100.9%	*p* = 0.820
		L	21.2 ± 4.5	20.8 ± 5.0	101.9%	*p* = 0.427
	Ball of Thumb	R	4.9 ± 1.3	6.9 ± 1.4	71.0%	*p* < 0.001 *
		L	4.9 ± 1.3	6.9 ± 1.3	71.0%	*p* < 0.001 *
	Thumb Press	R	3.7 ± 1.3	5.0 ± 1.4	74.0%	*p* < 0.001 *
		L	3.6 ± 1.4	4.8 ± 1.4	75.0%	*p* < 0.001 *
	Lateral Pinch	R	5.3 ± 1.1	5.4 ± 1.2	98.1%	*p* = 0.439
		L	4.8 ± 1.0	5.0 ± 1.1	96.0%	*p* = 0.165
	Palmar Pinch	R	4.9 ± 1.0	5.3 ± 1.3	92.5%	*p* = 0.010 *
		L	4.6 ± 0.9	5.0 ± 1.1	92.0%	*p* = 0.001 *

Note: ^1^: Student’s *t* test. *: *p* < 0.05.

**Table 6:** Prediction models for different types of hand strength using demographic information.

Type of Movement	Regression Equation	Adjusted R^2^
Grip_R	Grip_R = −1.164 − 10.445 × (sex) + 0.234 × (height) + 0.117 × (weight)	0.598
Grip_L	Grip_L = 30.478 − 8.591 × (sex) + 0.421 × (weight) − 0.853 × (BMI) + 0.080 × (age)	0.586
Ball of Thumb_R	Log(Ball of Thumb_R) = 0.662 + 0.004 × (weight) − 0.105 × (sex)	0.486
Ball of Thumb_L	Log(Ball of Thumb_L) = 0.664 + 0.004 × (weight) − 0.102 × (sex)	0.461
Thumb Press_R	Log(Thumb Press_R) = 0.696 + 0.003 × (weight) − 0.149 × (sex)	0.418
Thumb Press_L	Log(Thumb Press_L) = 0.694 + 0.003 × (weight) − 0.153 × (sex)	0.407
Lateral Pinch_R	Lateral Pinch_R = −1.293 − 1.681 × (sex) + 0.035 × (weight) + 0.018 × (age) + 0.029 × (height)	0.567
Lateral Pinch_L	Lateral Pinch_L = 5.434 − 1.712 × (sex) + 0.042 × (weight) + 0.01 × (age)	0.551
Palmar Pinch_R	Palmar Pinch_R = 6.675 − 1.532 × (sex) + 0.022 × (weight)	0.399
Palmar Pinch_L	Palmar Pinch_L = 6.210 − 1.426 × (sex) + 0.021 × (weight)	0.434

Note: Age: years; Sex: male = 1, female = 2; Weight: kg; Height: cm.

**Table 7:** Strength prediction models for different types of hand movement stratified by hand using demographic and anthropometric data, and opposite hand strength of the same hand movement.

Type of Movement	Regression Equation	Adjusted-R^2^
Grip_R	Grip_R = 5.397 + 0.944 × (Grip_L) − 2.472 × (sex) + 0.44 × (education)	0.951
Grip_L	Grip_L = −0.460 + 0.826 × (Grip_R) + 0.042 × (weight) + 0.31 × (age)	0.900
Ball of Thumb_R	Log(Ball of Thumb_R) = 0.089 + 0.863 × (Log(Ball of Thumb_L)) + 0.001 × (weight) − 0.017 × (sex)	0.889
Ball of Thumb_L	Log(Ball of Thumb_L) = 0.047 + 0.942 × (Log(Ball of Thumb_R))	0.883
Thumb Press_R	Log(Thumb Press_R) = 0.131 + 0.864 × (Log(Thumb Press_R)) − 0.023 × (sex)	0.879
Thumb Press_L	Log(Thumb Press_R) = 0.062 + 0.933 × (Log(Thumb Press_L)) − 0.017 × (sex)	0.877
Lateral Pinch_R	Lateral Pinch_R = 1.989 + 0.886 × (Lateral Pinch_L) − 0.485 × (sex)	0.856
Lateral Pinch_L	Lateral Pinch_L = −0.099 + 0.807 × (Lateral Pinch_R) + 0.012 × (weight)	0.853
Palmar Pinch_R	Palmar Pinch_R = −2.002 + 0.921 × (Palmar Pinch_L) + 0.017 × (height)	0.773
Palmar Pinch_L	Palmar Pinch_L = 2.046 + 0.698 × (Palmar Pinch_R) − 0.444 × (sex)	0.784

Note: Age: years; Sex: male = 1, female = 2; Weight: kg; Height: cm.

**Table 8:** Proposed prediction models for five different types of strength in the right hand using different types of strength in the right hand and demographic information.

Type of Movement	Regression Equation	Adjusted R^2^
Grip_R (kg)	Grip_R = −12.897 + 2.759 × (Lateral Pinch_R) − 5.609 × (sex) + 0.198 × (height)	0.708
	Grip_R = −11.756 − 2.810 × (Palmar Pinch_R) − 6.601 × (sex) + 0.185 × (height) − 0.064 × (weight)	0.704
Lateral Pinch_R (kg)	Lateral Pinch_R = 3.836 + 0.098 × (Grip_R) + 0.025 × (weight) − 0.740 × (sex) − 0.189 × (education)	0.679
	Lateral Pinch_R = 2.057 + 0.667 × (Palmar Pinch_R) − 0.930 × (sex) + 0.026 × (weight) + 0.009 × (age)	0.749
Palmar Pinch_R (kg)	Palmar Pinch_R = 3.564 + 0.097 × (Grip_R) − 0.402 × (sex)	0.559
	Palmar Pinch_R = 2.158 + 0.621 × (Lateral Pinch_R) − 0.278 × (sex)	0.654
Ball of Thumb_R (kg)	Log(Ball of Thumb_R) = 0.071 + 0.005 × (Grip_R) + 0.004 × (weight) + 0.002 × (height)	0.497
	Log(Ball of Thumb_R) = 0.190 − 0.636 × (Log(Thumb Press_R)) + 0.002 × (weight)	0.772
Thumb Press_R (kg)	Log(Thumb Press_R)= −0.074 + 0.007 × (Grip_R) + 0.002 × (weight) + 0.002 × (height)	0.436
	Log(Thumb Press_R) = 0.099 + 0.903 × (Log(Ball of Thumb_R)) − 0.054 × (sex) − 0.001 × (weight)	0.741

Note: Force: kgw; Age: years; Sex: male = 1, female = 2; Weight: kg; Height: cm.

## Data Availability

The data presented in this study are available on request from the corresponding author. The data are not publicly available due to privacy or ethical.

## Supplementary Materials

The following are available online at https://www.mdpi.com/1660-4601/18/1/187/s1, Table S1: Repeated-measures ANOVA of the strength of grip, lateral pinch and palmar pinch and age group, Table S2: Repeated-measures ANOVA of the strength of the ball of the thumb and thumb press and age group. Table S3: Person’s correlation coefficients between five types of hand strengths, demographic information, and anthropometric measures.

## Abbreviations

MSDs	Musculoskeletal disorders
CTS	Carpal tunnel syndromes
BMI	body mass index
CV	Coefficients of variation
ASHT	The American Society of Hand Therapists
Adjusted-R^2^	adjusted R-square
