# Supplementary material for: Normative Hand Strength of Healthcare Industry Workers in Central Taiwan
Source: Int J Environ Res Public Health. 2020 Dec 29;18(1):187. doi: 10.3390/ijerph18010187 (PMC7795098; doi:10.3390/ijerph18010187)
Supplement: Supplementary file 1 [file ijerph-18-00187-s001.pdf]

Supplementary Material

Table S1. Results of Repeated-measures ANOVA of the hand strength of grip, lateral pinch and palmar pinch and age group.

Sex	Hand Laterality	Source of variables	SS	df	MS	F	p-Value	Partial η^2
Male	Right	Within Subject						
		Type of Movement	89957.794	1.067	84307.565	2395.136	p < 0.001**	0.938
		Type of Movement*Age group	661.004	4.268	154.872	4.4	p = 0.002*	0.101
		Error	5896.691	167.522	35.199			
		Between Subject						
		Age	711.538	4	177.885	5.494	p < 0.001**	0.123
		Error	5083.277	157	32.378			
	Left	Within Subject						
		Type of Movement	78080.086	1.062	73493.364	2370.107	p < 0.001**	0.98
		Type of Movement*Age group	536.837	4.25	126.325	4.074	p = 0.003*	0.094
		Error	5172.161	166.798	31.008			
		Between Subject						
		Age	668.599	4	167.15	5.921	p < 0.001**	0.131
		Error	4431.899	157	28.229			
Female	Right	Within Subject						
		Type of Movement	31582.991	1.081	299229.689	2513.176	p < 0.001**	0.939
		Type of Movement*Age group	33.325	4.322	7.71	0.663	p = 0.630	0.016
		Error	2035.848	175.043	11.631			
		Between Subject						
		Age	53.345	4	13.336	1.082	p = 0.367	0.026

	Error	1996.087	162	12.322			
Left	Within Subject						
	Type of Movement	30373.008	1.072	15186.504	2833.257	p < 0.001**	0.946
	Type of Movement*Age group	80.41	4.288	18.751	1.875	p = 0.112	0.044
	Error	1736.668	173.68	9.999			
	Between Subject						
	Age	114.951	4	28.738	2.669	p = 0.034*	0.062
	Error	1744.513	162	10.769			

Note: * $p < 0.05$; ** $p < 0.001$

Table S2. Results of Repeated-measures ANOVA of the hand strength of the ball of the thumb and thumb press and age group

Sex	Hand Lat-erality	Source of variables	SS	df	MS	F	p-Value	Partial η^2
Male	Right	Within Subject						
		Type of Movement	0.772	1	0.772	264.75	p < 0.001**	0.628
		Type of Movement*Age group	0.005	4	0.001	0.397	p = 0.810	0.01
		Error	0.458	157	0.003			
		Between Subject						
		Age	0.032	4	0.008	0.349	p = 0.845	0.009
		Error	3.586	157	0.023			
	Left	Within Subject						
		Type of Movement	0.814	1	0.814	234.911	p < 0.001**	0.599
		Type of Movement*Age group	0.008	4	0.002	0.554	p = 0.696	0.014
		Error	0.554	157	0.003			
		Between Subject						
		Age	0.05	4	0.013	0.485	p = 0.747	0.012
		Error	4.089	157	0.026			
Female	Right	Within Subject						
		Type of Movement	1.285	1	1.285	332.945	p < 0.001**	0.673
		Type of Movement*Age group	0.007	4	0.002	0.46	p = 0.765	0.011
		Error	0.625	162	0.004			
		Between Subject						
		Age	0.66	4	0.017	0.626	p = 0.664	0.015
		Error	4.274	162	0.026			

Left	Within Subject						
	Type of Movement	1.552	1	1.552	317.436	p < 0.001**	0.662
	Type of Movement*Age group	0.009	4	0.002	0.453	p = 0.770	0.011
	Error	0.792	162	0.005			
	Between Subject						
	Age	0.032	4	0.008	0.349	p = 0.845	0.028
	Error	3.586	157	0.023			

Note: * $p < 0.05$; ** $p < 0.001$

Table S3. Person's correlation coefficients between hand strengths, demographic information, and anthropometric measures.

Variables	1	2	3	4	5	6	7	8	9	10	11	12	13	14	15	16	17	18	19	20	21
1. Gender	1	0.028	-.728**	-.557**	-.260**	-.614**	-.653**	-.155**	0.06	-.126*	-.242**	-.743**	-.721**	-.610**	-.593**	-.614**	-.607**	-.700**	-.684**	-.611**	-.637**
2. Age	0.028	1	.206**	-0.017	.117*	0.09	0.048	0.089	-0.078	-.516**	.163**	0.003	0.045	-0.007	0.008	0.015	0.05	0.077	0.056	0.04	0.046
3. Height	-0.728**	-0.206**	1	.628**	.169**	.585**	.648**	.144**	0.023	.234**	.212**	.669**	.654**	.573**	.563**	.539**	.534**	.610**	.594**	.529**	.525**
4. Weight	-0.557**	-0.017	0.628**	1	.868**	.601**	.579**	.116*	-0.079	0.082	0.1	.568**	.580**	.625**	.609**	.517**	.508**	.604**	.618**	.481**	.503**
5. BMI	-0.260**	0.117*	0.169**	0.868**	1	.394**	.329**	0.067	-.109*	-0.059	-0.001	.302**	.326**	.436**	.425**	.324**	.317**	.386**	.413**	.280**	.307**
6. Hand width	-0.614**	0.09	0.585**	0.601**	0.394**	1	.647**	.137*	0.011	0.032	.188**	.575**	.562**	.485**	.485**	.454**	.474**	.538**	.543**	.465**	.464**
7. Hand_span	-0.653**	0.048	0.648**	0.579**	0.329**	0.647**	1	.317**	-0.051	0.083	.199**	.631**	.630**	.512**	.477**	.438**	.438**	.553**	.556**	.477**	.508**
8. Grip diameter	-0.155**	0.089	0.144**	0.116*	0.067	0.137*	0.317**	1	-0.036	-0.004	.131*	.113*	.134*	.115*	0.094	.138*	.120*	.153**	.141*	.132*	.163**
9. Dominant hand	0.06	-0.078	0.023	-0.079	-0.109*	0.011	-0.051	-0.036	1	0.012	-0.06	0.029	-0.036	0.042	0.002	0.021	0.036	-0.031	-.117*	-0.002	-0.083
10. Education	-0.126*	-0.516**	0.234**	0.082	-0.059	0.032	0.083	-0.004	0.012	1	-0.02	.128*	0.09	0.085	0.078	0.083	0.055	0.026	0.033	0.067	0.051
11. Exercise	-0.242**	0.163**	0.212**	0.1	-0.001	0.188**	0.199**	0.131*	-0.06	-0.02	1	.211**	.231**	0.106	0.106	.142**	.148**	.157**	.178**	.158**	.179**
12. Grip_R	-0.743**	0.003	0.669**	0.568**	0.302**	0.575**	0.631**	0.113*	0.029	0.128*	0.211**	1	.947**	.621**	.625**	.637**	.635**	.790**	.790**	.744**	.762**

13. Grip_L	-0.721**	0.045	0.654**	0.580**	0.326**	0.562**	0.630**	0.134*	-0.036	0.09	0.231**	0.947**	1	.585**	.599**	.605**	.615**	.777**	.793**	.724**	.769**
14. Ball of Thumb_R	-0.610**	-0.007	0.573**	0.625**	0.436**	0.485**	0.512**	0.115*	0.042	0.085	0.106	0.621**	0.585**	1	.940**	.852**	.831**	.615**	.601**	.590**	.552**
15. Ball of Thumb_L	-0.593**	0.008	0.563**	0.609**	0.425**	0.485**	0.477**	0.094	0.002	0.078	0.106	0.625**	0.599**	0.940**	1	.833**	.824**	.600**	.598**	.571**	.552**
16. Thumb Press_R	-0.614**	0.015	0.539**	0.517**	0.324**	0.454**	0.438**	0.138*	0.021	0.083	0.142**	0.637**	0.605**	0.852**	0.833**	1	.936**	.620**	.598**	.583**	.565**
17. Thumb Press_L	-0.607**	0.05	0.534**	0.508**	0.317**	0.474**	0.438**	0.120*	0.036	0.055	0.148**	0.635**	0.615**	0.831**	0.824**	0.936**	1	.616**	.610**	.570**	.565**
18. Lateral Pinch_R	-0.700**	0.077	0.610**	0.604**	0.386**	0.538**	0.553**	0.153**	-0.031	0.026	0.157**	0.790**	0.777**	0.615**	0.600**	0.620**	0.616**	1	.921**	.808**	.801**
19. Lateral Pinch_L	-0.684**	0.056	0.594**	0.618**	0.413**	0.543**	0.556**	0.141*	-0.117*	0.033	0.178**	0.790**	0.793**	0.601**	0.598**	0.598**	0.610**	0.921**	1	.765**	.834**
20. Palmer Pinch_R	-0.611**	0.04	0.529**	0.481**	0.280**	0.465**	0.477**	0.132*	-0.002	0.067	0.158**	0.744**	0.724**	0.590**	0.571**	0.583**	0.570**	0.808**	0.765**	1	.876**
21. Palmer Pinch_L	-0.637**	0.046	0.525**	0.503**	0.307**	0.464**	0.508**	0.163**	-0.083	0.051	0.179**	0.762**	0.769**	0.552**	0.552**	0.565**	0.565**	0.801**	0.834**	0.876**	1

Note: * $p < 0.05$; ** $p < 0.001$
